# *Olea europaea* small RNA with functional homology to human miR34a in cross-kingdom interaction of anti-tumoral response

**DOI:** 10.1038/s41598-018-30718-w

**Published:** 2018-08-17

**Authors:** Antonella Minutolo, Marina Potestà, Angelo Gismondi, Stefano Pirrò, Marco Cirilli, Fabiano Gattabria, Andrea Galgani, Libera Sessa, Maurizio Mattei, Antonella Canini, Rosario Muleo, Vittorio Colizzi, Carla Montesano

**Affiliations:** 1 Department of Biology, University of Rome “Tor Vergata”, Rome, Italy; 2 Mir-Nat s.r.l, Rome, Italy; 3 Department of Agricultural and Forestry, Science, University of Tuscia, Viterbo, Italy; 4 Interdepartmental Center for Animal Technology, University of Rome “Tor Vergata”, Rome, Italy

## Abstract

Functional foods include compounds with nutritional and health properties. The human diet could play a stronger role in cancer prevention. Only a few studies have described the presence of plant small RNA, in humans who were fed with plant foods, which demonstrated the ability of these molecules to modulate consumer’s genes and evidenced the existence of a plant-animal regulation. Through *in silico* prediction, *Olea europaea* small RNAs (sRs), which had been previously reported as miRNAs, were identified, each with functional homology to *hsa*-miR34a. According to this initial funding, we investigated the ability of *oeu*-sRs to regulate tumorigenesis in human cells. The transfection of these synthetic *oeu*-sRs reduced the protein expression of *hsa*-miR34a mRNA targets, increased apoptosis and decreased proliferation in different tumor cells; by contrast, no effect was observed in PBMCs from healthy donors. The introduction of *oeu*-small RNA in *hsa*-miR34a-deficient tumor cells restores its function, whereas cells with normal expression of endogenous *hsa*-miR34a remained unaffected. The natural *oeu*-small RNAs that were extracted from *O*. *europaea* drupes induce the same effects as synthetic sRs. Careful research on the small RNA sequences executed for mapping and annotation in the genome of *O*. *europaea* var. Sylvestris and var. Farga led to the hypothesis that RNA fragments with functional homology to human miRNAs could be generated from the degradation of regions of RNA transcripts. These results indicate the possibility of developing novel natural non-toxic drugs that contain active plant-derived tumor-suppressing small RNA with functional homology to *hsa*-miRNAs and that can support antineoplastic strategies.

## Introduction

Small RNAs, including microRNAs (miRNAs), are a class of small (19–24 nucleotides) non-coding RNAs that post-transcriptionally regulate gene expression through interacting with specific mRNAs. In all species, miRNA-mediated gene regulation may occur through either degradation or post-translation blocking of mRNAs. In plants, miRNAs need high complementarity to recognize their substrate, while the target cleavage is considered the predominant pathway to repress gene expression. In animals, the semi-complementary interaction leads to silencing achieved via translational repression^1^.

In humans, miRNAs (*hsa*-miRNAs) have been strongly associated with the regulation of critical biological events, including development, differentiation, inflammation, apoptosis and carcinogenesis^2,3^. The miRNA34 family (*hsa-*miR34) is an important class of human onco-suppressor miRNAs: the expression of its components is frequently repressed in tumors, compared with normal tissues^4,5^.

Bioinformatics and *in vitro* studies indicate that *hsa-*miR34a is involved in different apoptotic cellular mechanisms by repressing *B-cell lymphoma 2* (*BCL2*) mRNA translation. BCL2 is a crucial anti-apoptotic protein that is involved in the intrinsic pathway; it blocks NAD-dependent deacetylase sirtuin-1 (SIRT1), which induces apoptosis via regulating p53 activity^6–9^. In hepatocellular carcinoma (HCC), *hsa-*miR34a may act as a tumor suppressor miRNA that inhibits cell growth, migration and invasion and regulates the epithelial to mesenchymal transition (EMT) through SNAIL modulation^10,11^.

Evidence has been found for the therapeutic application of *hsa*-miR34 in murine tumor models of lung, liver, prostate and lymphoma. In such systems, systemic delivery of nanoparticles loaded with synthetic *hsa-*miR34a mimics showed robust tumor inhibition^12–14^. An *hsa-*miR34a-based therapy is currently in Phase I clinical trials^15,16^.

The majority of the human population worldwide has long used medicinal plants as their primary source of health care. Many of these medicinal plants may have the scientific evidence to be considered in general practice^17^.

Recently, scientific studies have demonstrated the existence of a so-called “cross-kingdom interaction”, which is mediated by exogenous miRNAs that are derived from plants: these, inside the host cell, serve to regulate the gene expression machinery^18^.

Among them, of interest is the research by Zhou and colleagues, who identified the first bioactive compound in *Traditional Chinese Medicine*: miR2911 from honeysuckle. This compound is able to target various Influenza A viruses (IAVs), and it represents a novel type of natural product with effective anti-viral activity^19^.

Other studies have reported that miRNAs derived from plant foods are also functional in mammals, and they regulate the expression of host genes. Zhang and colleagues described the presence of rice miRNAs in the serum of humans and animals fed with the same foodstuff ^20^.

In particular, rice miRNA168a was able to bind the mRNA of human and mouse low-density lipoprotein receptor adapter protein 1 (LDLRAP1) and inhibit its expression in the liver, consequently decreasing LDL removal from the plasma^20^. Similarly, Liang *et al*. demonstrated the survival of miR172 from *Brassica oleracea* in the tissues and feces of mice fed with this plant species^21^.

In another study, through analysis of publicly available information, Liu *et al*.^22^ identified abundant plant miRNA sequences via sequencing of plasma small RNAs; plant miRNA 2910, which is homologous to *hsa*-miR4259 and *hsa*-miR4715-5p, was found in high levels in plasma samples and was predicted to target the human JAK-STAT signaling pathway mRNA SPRY4.

Subsequent studies have shown that exogenous plant miRNAs that are ingested in food persist for several hours in the mammalian gut and rapidly move into the bloodstream. Moreover, plant miRNAs have been demonstrated to be unaffected by heat treatment, due to cooking, and by digestive processes^23,24^.

Using several computational approaches to predict miRNA-mRNA interactions^25–29^, some *Moringa oleifera* miRNAs that exhibit putative functional homology to their mammalian counterparts were identified, and their ability to regulate human target genes in cell-transfection assays was also demonstrated in a specific cell line^30^.

In the current work, olive (*Olea europaea* L.) small RNA, previously reported as miRNAs^31^, were predicted to show functional homology to various human microRNAs; additionally, their potential human mRNA targets were identified. Taking into consideration the use of synthetic *hsa-*miR34a as an anti-tumoral molecule in different preclinical and clinical trials^4,5,12,13^, we selected the putative novel olive miRNAs (*oeu*-miR20, *oeu*-miR27 and *oeu*-miR34, according to the nomenclature assigned by Yanik *et al*.)^31^ with predicted homology to *hsa-*miR34a. Therefore, the purpose of this research focused on assessing the ability of olive small RNAs (sRs) to regulate tumorigenesis.

A series of transfection experiments were performed on lymphoid, monocytoid and hepatic cell lines and PBMCs from healthy donors to validate the effective ability of synthetic sequences of *oeu*-sRs to modulate the protein expression of *hsa-*miR34a-specific targets (*SIRT1*, *BCL2* and *SNAIL*) and, subsequently, the down-streamed biological effect of such treatments.

## Results

### Identification of *Olea europaea* miRNAs that are homologous to human miRNAs and their target genes and evaluation of the free energy of duplex formation

**Figure 1:**
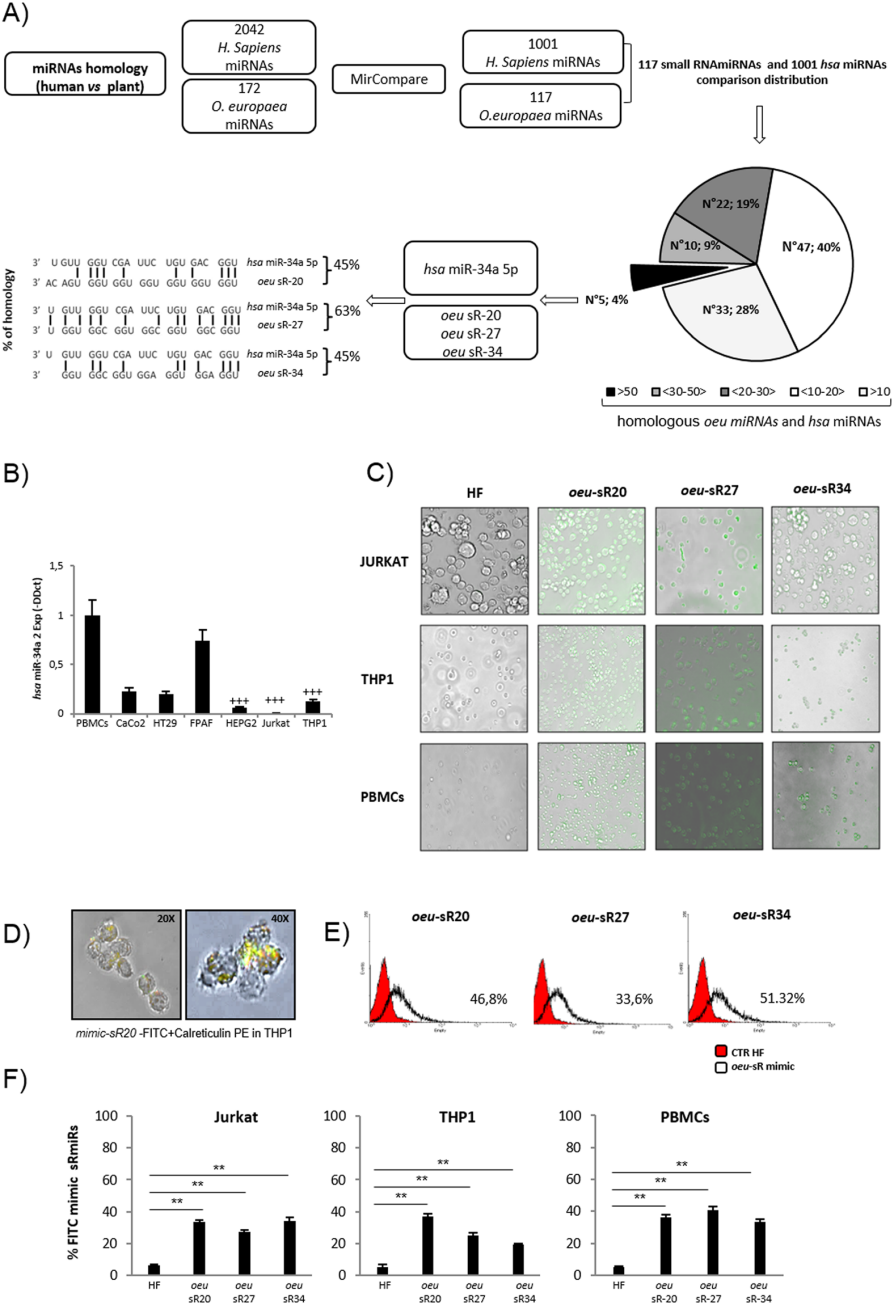
Bioinformatics analysis and transfection efficiency of synthetic FITC-*oeu*-sR20, -27 and -34 in Jurkat, THP1 cell lines and in PBMCs from healthy donors. Workflow of the MirCompare analysis used for comparing *H*. *sapiens* microRNA and *O*. *europaea* putative miRNAs (panel A): Sequences homology comparison (n = 117 *oeu-human* miRNAs homologous) and distribution analysis. Among the 5 *oeu-*sRs having more than 50 homologous human miRNAs, *oeu*-sR20, -27 and -34 (homologous to *hsa*-miRNA34a) were aligned and the homology percentage was calculated. qRT-PCR expression analysis (ΔΔCT method) of *hsa*-miR34a in cell lines compared to PBMCs; data are expressed as means ± SD of three independent samples (panel B). Quantification was performed using the threshold cycle (Ct) comparative method and normalized with 18S rRNA. Presence of fluorescinated *oeu*-sRs in Jurkat, THP-1 cell lines and PBMCs 72 hours after transfection (panel C). The efficiency of *oeu*-sRs transfection was confirmed by observing the fluorescent cells as they appear at fluorescence microscope in these representative samples (Evos Floid Cells Imaging Station Life Technologies). Localization of *oeu*-sR20 FITC and calreticulin (ER) in THP1 cell after 72 hours from mimic *oeu-*sR20 transfection (panel D); Representative histogram obtained by flow cytometric analysis of fluorescinated *oeu*-sRs positive cells in Jurkat cell line after 72 hours from miRNA transfection (panel E). Red curves represent control cells receiving only HF, the empty curves represent transfected cells. The percentages of FITC-miRNAs positive Jurkat, THP1 cells and PBMCs were assessed via Cytexpert 2.0 software (Beckman Coulter) and showed in graphs (panels F). The results show the means ± SD of three independent experiments and 10 PBMCs samples (mean of percentage FL-1-positive for all three *oeu*-sR vs HF) 72 hours after from miRNA transfection.

MirCompare^32^ was used to compare the data set of miRNAs from *Homo sapiens*^33^ and *O*. *europaea*^31^ (Fig. 1A). The analysis of 172 *O*. *europaea* and 2042 *H*. *sapiens* microRNAs generated a total of 351,224 different comparisons. The cut-off value of r lower than 0.5 made it possible to reduce the number to 12,134. After the second filtering phase over the seed region, only 2,164 different comparisons were obtained. These comparisons analyze 117 *O*. *europaea* putative microRNAs and 1,001 *H*. *sapiens* microRNAs. The complete MirCompare analysis can be found in the Supplementary Information (Table S1).

Clustering analysis on the abundance distribution made it possible to generate 5 different bins (Fig. 1A), with different degrees of sequence homology: 1) each of the 33 out of n. 117 (28%) plant putative miRNAs were homologous with less than 10 human miRNAs; 2) n. 47 (40%) plant putative miRNAs exhibited homology with human homologous miRNAs ranging between 10 and 20; 3) n. 22 (19%) plant putative microRNAs showed a sequence homology between 20 and 30 human miRNAs; 4) n. 9 (10%) had at least 30 homologous human miRNAs; and 5) only n. 5 out 117 putative *oeu*-microRNAs had a number of homologous human miRNAs higher than 50.

Among this last group of putative *O*. *europaea* miRNAs, we selected the putative novel miRNAs, *oeu*-miR20, *oeu-*miR27 and *oeu-*miR34, which showed sequence homology (45%, 63% and 45%, respectively) with *hsa*-miRNA34a-5p, an important regulator of tumor suppression^4,5,12,13^ (Fig. 1A). However, a zero alignment hit was obtained from the analysis of validation of these putative novel *oeu*-miR sequences that was performed using miRDeep2^34^ in *O*. *europaea* var. sylvestris. Therefore, a wide bioinformatics analysis (Table S2) was performed to verify whether the sequences were true-to-type miRNAs and for mapping and annotating sequences on the genomes of *O*. *europaea* var. Sylvestris and var. Farga.

As a result of the bioinformatics analysis (Table S2) that was conducted to verify whether the sequences were true-to-type miRNAs, we have redefined the concept of small RNA miRNA-like in the acronym sRs, without changing the numbering reported by Yanik *et al*.^31^. Therefore, in the text, from now on, each small RNA from Olea will be reported as sRs instead of miR.

The interaction of *oeu*-sRs with human mRNAs was studied via transfecting synthetic (mimics) *oeu*-sR20, *oeu*-sR27 and *oeu*-sR34 or *hsa*-miR34a into Jurkat lymphoid and into THP1 monocytoid cell lines, as well as into PBMCs from healthy donors.

THP1 monocytoid, Jurkat E6-1 lymphoid cell lines and HEPG2 hepatoma cells were selected for their low levels of human miR34a compared to other human tumor cell lines (HT29, CaCo-2, FPAF) and PBMCs from healthy donors (Fig. 1B).

The *oeu*-sRs transfection efficiency was evaluated in Jurkat and THP-1 cell lines and PBMCs by using fluorescent mimics via the EVOS FLoid cell imaging station and by monitoring the percentage of fluorescent-positive (FL1-positive) cells via flow cytometric analysis (Cytoflex, Beckman Coulter).

Seventy-two hours after transfection, both cell lines and PBMCs were efficiently transfected with the three *oeu*-sR mimics (*oeu*-sR20, *oeu*-sR27 and *oeu*-sR34), as shown by green fluorescence (Fig. 1C).

In one representative picture (Fig. 1D), the FITC *oeu*-sR20 in THP1 cells co-localized with calreticulin, a specific endoplasmic reticulum (RE) molecule, thereby suggesting the ability of plant sRs to localize in the specific cellular compartment where endogenous miRNAs operate.

Cell lines and PBMCs treated with High Fect vehicle control (HF) showed a mean intensity fluorescence (MIF) that was lower than 10^2^ (Fig. 1E, representative flow cytometric analysis in Jurkat cells). Cells were considered FL1-positive when the MIF was higher than 10^3^. Based on this threshold, the percentage of FITC-positive Jurkat and THP1 cells, as well as PBMCs, was significantly higher for all *oeu*-sRs than for HF control cells. This demonstrates the possibility of transfecting plant sR mimics into human cell lines (Fig. 1E,F).

### The *oeu*-sR mimics inhibit SIRT-1 and BCL-2 protein expression likewise to *hsa*-miR34a

The COMIR^29^ and Diana Tarbase^35^ algorithms were used to identify human genes potentially regulated by *O*. *europaea* sRs, and free energy variation of each sR:mRNA duplex formation was assigned. *Oeu*-sR20, *oeu*-sR27 and *oeu*-sR34 show a high propensity to bind *SIRT1*, *BCL2* and *SNAIL* transcripts, which proved similar or superior to that of the human homologous *hsa*-miR34 (Table 1).

**Table 1:** Free Energy variation (ΔG) in *hsa*-miRNA-mRNA and *oeu*-sR-mRNA duplex formation.

	*hsa*-miR-34a-5p	*oeu*-sR20	*oeu*-sR27	*oeu*-sR34
*BCL2*	−26,94	−37,52	−74,27	−27,02
*SIRT1*	−32,88	−26,00	−28,11	−29,35
*SNAIL*	−18,97	−25,46	−26,41	−59,57

**Figure 2:**
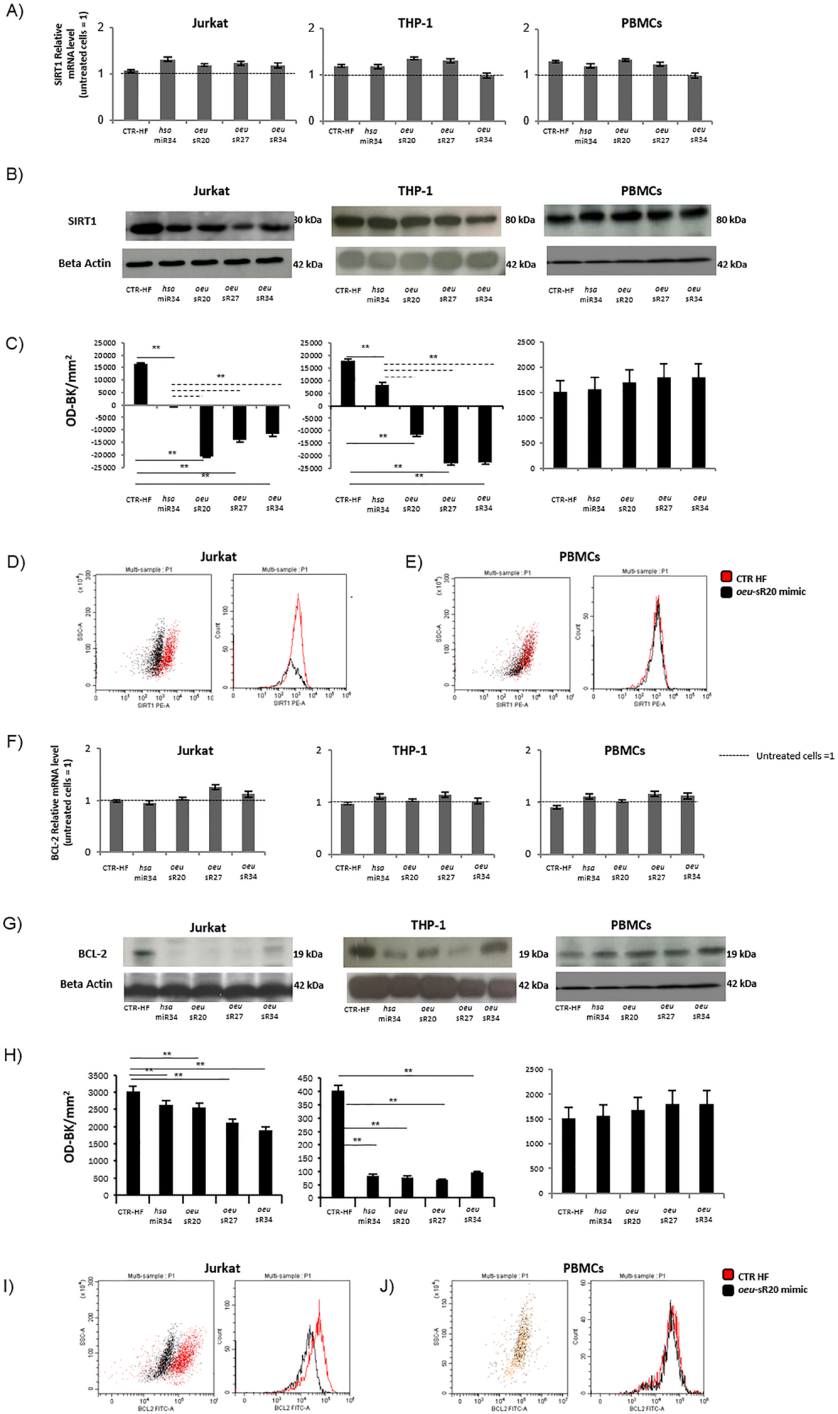
Effects of *oeu*-sRs transfection on modulation of *SIRT1* and *BCL2* mRNA and protein in Jurkat and THP-1 cell lines and in PBMCs from healthy donors. Relative qRT-PCR expression analysis of *SIRT1* and *BCL2* genes at 72 hours after *oeu*-sRs and *hsa*-miR34a transfection (panel A and F). The analysis was carried out on three independent biological experiments and expressed as a fold change in respect to untreated samples previously normalised with a housekeeping gene (beta actin). Western Blot assay of SIRT1 and BCL-2 proteins modulation after *oeu-*sRs transfection (panel B and G, Figures S1 and S2). One representative of three independent biological experiments is reported and the reference beta actin protein was detected. Western blot analysis of SIRT1 and BCL-2 protein expression was quantified by densitometric analysis (panels C and H). Each sample was normalised to its respective beta actin value and the background (Bkg) of the picture was subtracted. Values were expressed as OD-Bkg/mm^2^ (means ± SD of at least three independent experiment, p < 0.05 treated vs HF control). Flow cytometry analysis of SIRT1 (panels D and E) and BCL-2 (panels I and J) proteins intracellular expression in Jurkat and PBMcs 72 hours after *oeu* miR20 like transfection. One representative dot plot overlay, and histogram overlay of three independent biological experiments is reported.

The transfection of the mimics *oeu-*sR20, *oeu*-sR27 and *oeu*-sR34 did not affect the transcript levels of the *SIRT1* and *BCL2* genes in both THP1 and Jurkat cell lines and in PBMCs (Fig. 2A and F). However, the same cells exhibited a significant decrease in SIRT1 (Fig. 2B,C) and BCL2 proteins (Fig. 2G,H), compared with HF-treated cells (p < 0.05 for all treatment *vs* HF cells and vs *hsa*-miR34a). These results are consistent with the mechanism mediated by the post transcriptional function of miRNAs. The modulation of SIRT1 and BCL2 protein expression after transfection was also confirmed via flow cytometry assay (Fig. 2D,E,I,J). In contrast, the transfection of sRs in PBMCs from healthy donors exhibited no effect on the modulation of SIRT1 and BCL2 proteins. Transfection with an *hsa-*miR34a mimic scramble exhibited no effect in all the cell lines tested (data not shown).

### *Oeu*-sRs-mimic transfection affects cell viability and apoptosis

**Figure 3:**
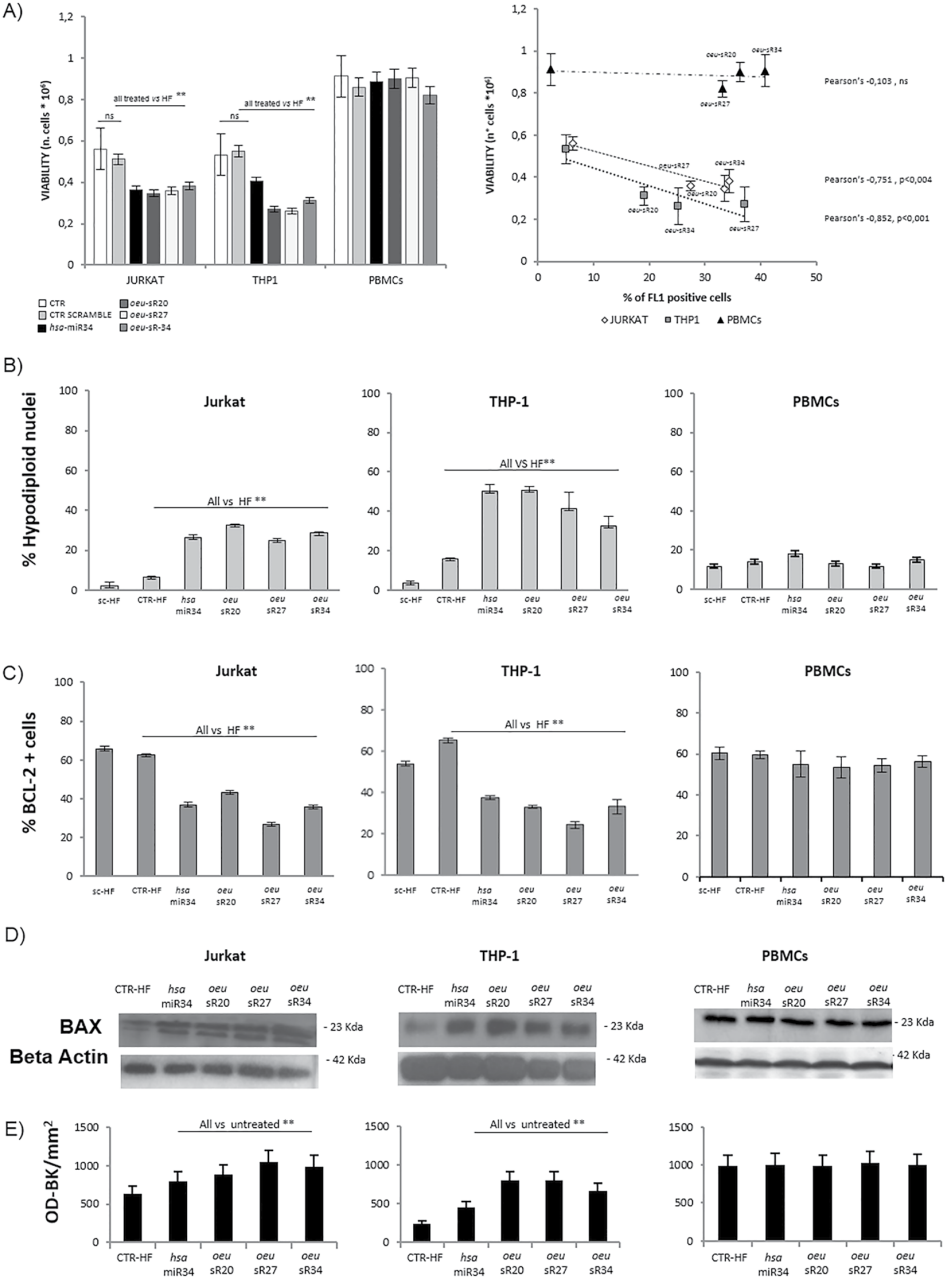
Effects of *oeu*-sR-20, -27 and -34 on cell viability and apoptosis in Jurkat, THP-1 cell lines and PBMCs from healthy donors. Cell viability (panel A) analysed via Trypan blue exclusion assay (left panel) and correlation between efficiency of transfection and cell viability (right panel). Percentage of apoptotic cells and percentage of BCL-2 positive cells after *oeu*-sRs and *hsa*-miR34a transfection analysed via Flow cytometry (panel B and C) (means ± SD at least three independent biological experiments performed, p < 0.05 treated vs control); one representative of the three independent biological experiments of Western Blotting assay of BAX modulation after *oeu*-sRs and *hsa*-miR34a transfection (panel D, Figure S3). In the densitometric analysis (panel E) (means ± SD) of three independent biological experiments, values were expressed as OD-Bkg/mm^2^. For all experiments, we show means ± SD of at least of three independent experiments performed, p < 0.05 treated vs control.

To assess whether the decrease in SIRT1 and BCL2 proteins via *oeu*-sR might also affect cell proliferation and apoptosis, the cell number and viability of THP1 and Jurkat cells were evaluated via the Trypan-blue-exclusion test at 72 hours after transfection. As shown in Fig. 3A (left panel), statistically significant differences (p < 0.05) were observed for all treatments *vs* High Fect (HF) (sample treated with lipofectamine only) cells. Moreover, a significant inverse correlation between the percentage of sR-positive cells and the cell count (Fig. 3A right panel) suggested a direct effect of *oeu*-sRs on host cell viability. No effect on cell viability was reported for sR-transfected PBMCs.

Jurkat cells that were treated with mimics of *oeu-*sR20, *oeu*-sR27, *oeu-*sR34 and *hsa*-miR34a showed a significant increase in hypodiploid nuclei, as assessed via propidium-iodide incorporation. This highlights apoptosis in cells exposed to *O*. *europaea* sRs compared with the control samples (Fig. 3B). The results obtained from THP1 analysis confirmed those observed in Jurkat cells, whereas in PBMCs from healthy donors, no significant difference was observed in the apoptosis levels.

To confirm the role of *oeu*-sR20, *oeu-*sR27 and *oeu-*sR34 in apoptosis mediated via the BCL2 pathway, the expression of intracellular BCL2 was analyzed through flow cytometry. The treatment of Jurkat and THP-1 cells with mimics derived from *oeu*-sR sequences induced a significant decrease of BCL2-positive cells compared with the HF control (Fig. 3C); this event was associated with a significant increase in the pro-apoptotic BAX protein level, as assessed via western blot analysis (Fig. 3D,E).

### Small RNA extracted from *O*. *europaea* drupes affects apoptosis and viability of Jurkat cells

**Figure 4:**
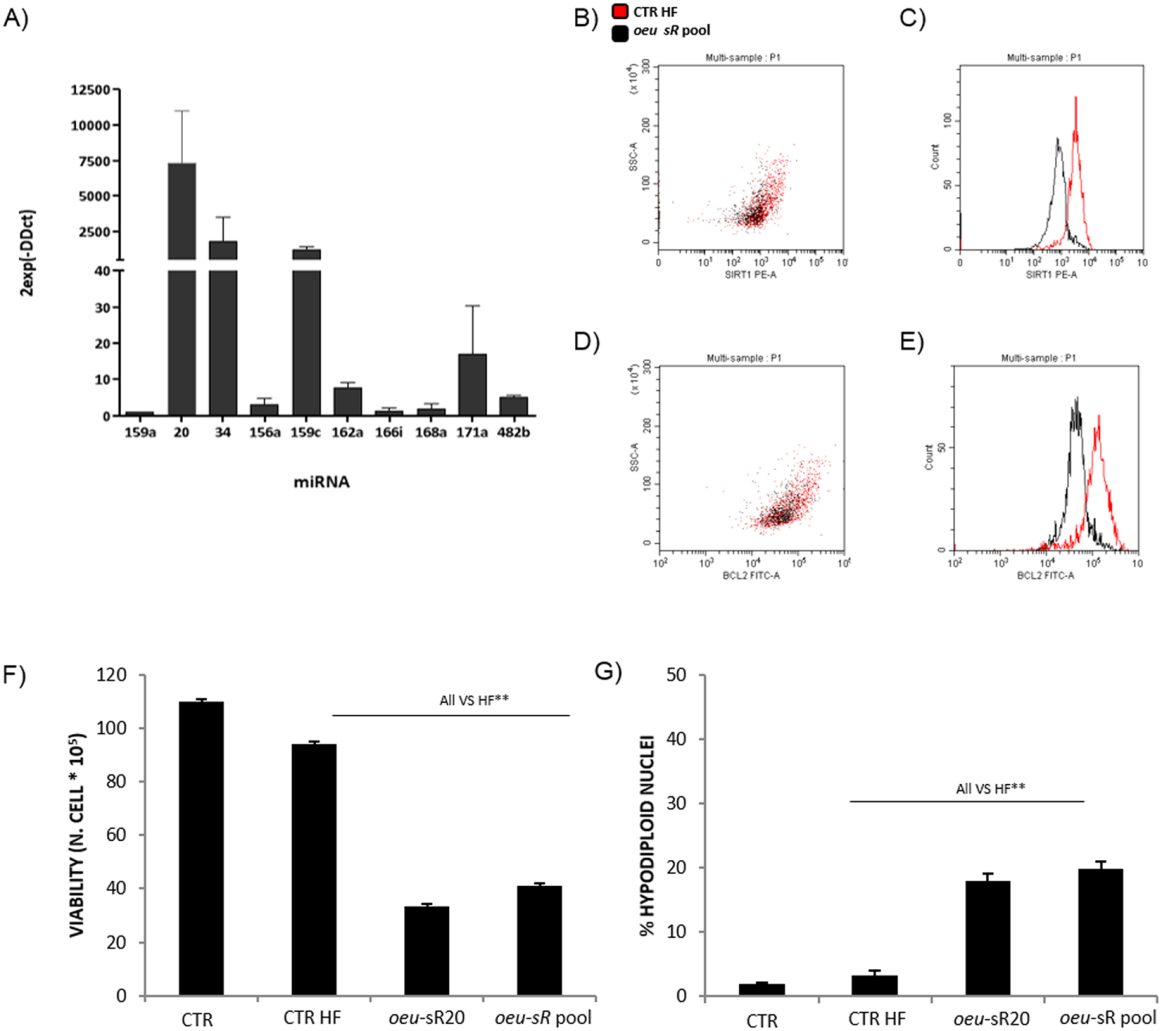
Effects of *oeu*-sR pool derived from *O*. *europaea* drupe. Relative RT-qPCR expression analysis of some *oeu* miRNAs contained in the pool of small RNAs extracted from drupes of *O. europaea* (panel A). The analysis was carried out on three independent biological experiments and expressed as fold change with respect to *oeu*-miR159 expression previously normalised with a housekeeping gene (5 S rRNA). Flow cytometry analysis of SIRT1 (panels B and C) and BCL2 (panels D and E) proteins intracellular expression in Jurkat 72 hours after *oeu*-sR pool transfection. One representative dot plot overlay, and histogram overlay of three independent biological experiments is reported (% of SIRT1 and BCL2 positive cells, *oeu*-sRs *vs* HF, SIRT1% 2.36 ± 0.36** vs 10.32 ± 0.63; BCL2% 42.63 ± 8.65 vs 70.51 ± 4.62**). Cell viability analysed via Trypan blue (panel F), percentage of apoptotic cells (panel G) after *oeu-*sRs or *oeu-sR*20 analysed via Flow cytometry (means ± SD of three independent biological experiments, p < 0.05 treated vs control).

The pool of small RNAs that were extracted from mature drupes of olive and analyzed via RT-qPCR revealed the presence of plant miRNAs and sRs. Among them, *oeu*-sR34 and *oeu*-sR20 were the most highly expressed (Fig. 4A). To confirm the activity of this olive-fruit-extracted pool on human cells, it was transfected in Jurkat cells, and its effect on SIRT1 and BCL2 protein expression were analyzed via flow cytometry.

The treatment of Jurkat with the *oeu*-sR pool induced a significant decrease in SIRT1- and BCL2-positive cells (Fig. 4B,D) and a significant decrease in MIF (Fig. 4C,E) compared with the HF control sample.

Cell viability and apoptosis were analyzed and compared to the pro-apoptotic synthetic *oeu*-sR20.

Jurkat cells treated with the *oeu*-sR pool showed a significant decrease in cell viability and increase in apoptosis, similar to the effects induced by the sR20 mimic (Fig. 4F,G).

### Transfection of *oeu*-sR20 mimic and *oeu*-sR pool in a hepatoma cell line reduces the expression of SNAIL

The impact of *oeu*-sRs on the gene expression of *SNAIL*, another specific target of *hsa*-miR34a, and the relative biological effects were analyzed in the HepG2 hepatoma cell line after *oeu*-sR20 or *oeu*-sR pool transfection. During tumorigenesis, the increase in SNAIL protein down-regulates the synthesis of E-cadherin, which induced the acquisition of metastatic potential during the late stages of epithelial tumor progression^36^.

**Figure 5:**
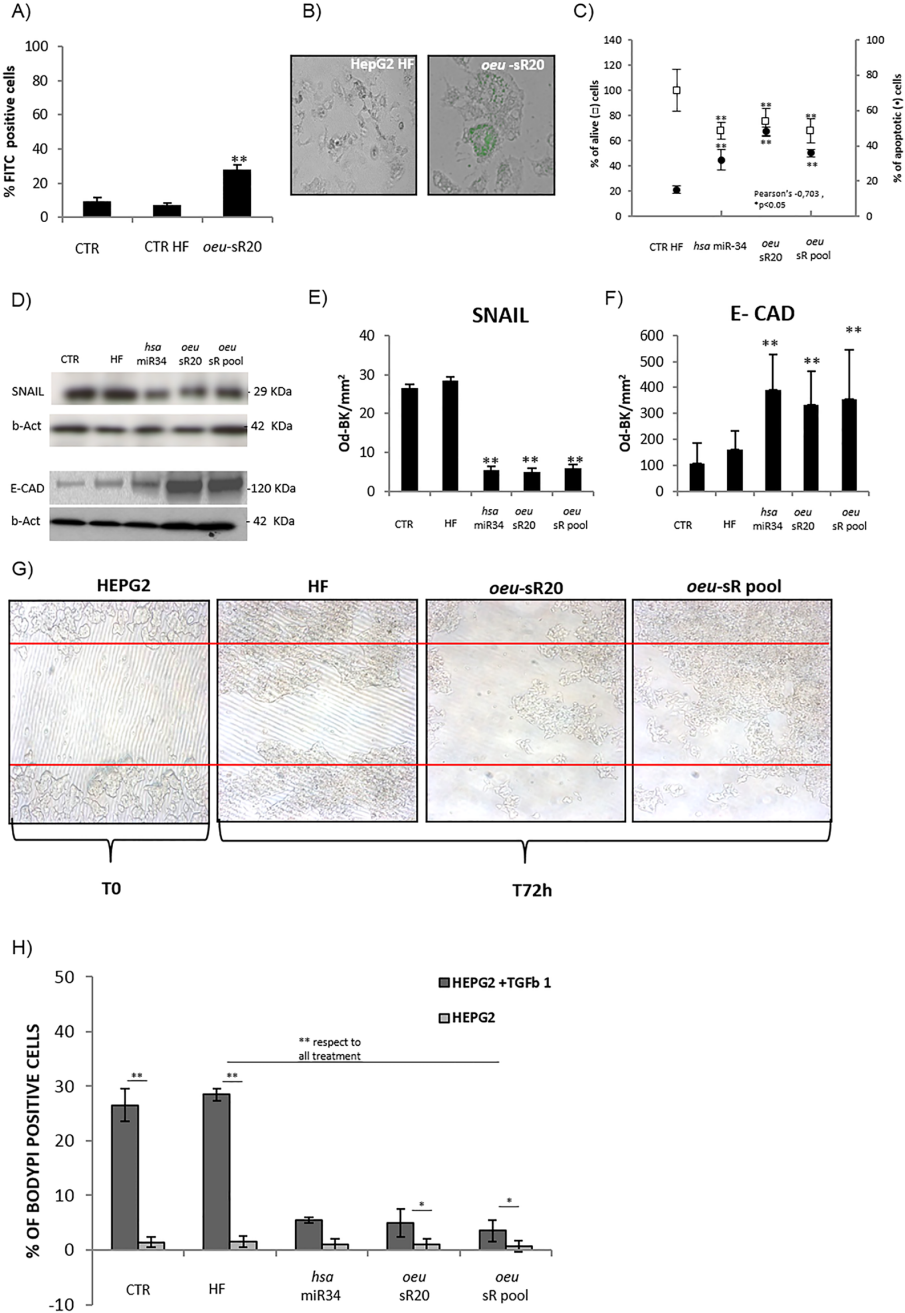
*oeu-*sR pool transfection effects on SNAIL protein modulation and lipid accumulation in Hepatoma cell lines. Percentage of FITC- *oeu*-sR20 positive HepG2 (panel A). The efficiency of miRNA transfection was confirmed by observing the fluorescent cells as they appear at fluorescence microscope in panel B. Percentage of viabilty and apoptosis in *oeu*-sR20, *hsa*-miR34 and *oeu*-sR pool transfected Hep-G2 (panel C). One representative western blot image of SNAIL and E-Cadherin (panel D, Figure S4) in HepG2 cells after transfection as well as densitometric analysis of three independent biological experiments are reported (panel E and F). The scratch test was used to detect HepG2 migration rates at 0 and 72 h after transfection. The representative microscopy picture (×40) was shown (panel G). FACS analysis of lipid accumulation induced by TGFbeta1 in HEPG2 in the presence or absence of *oeu*-sR20, *oeu*-sR pool and *hsa*-miR34, 72 hours post-transfection (panel H). Results are from at least three independent biological experiments. Histograms represent the mean and the bars ± S.D, **p < 0.01.

The transfection efficiency was significant in the hepatoma cell line (Fig. 5A,B), as demonstrated in Jurkat and THP1 cells; the transfection with the mimics (*oeu*-sR20 and *hsa-*miR34) resulted in a significant decrease in cell viability that correlated with the increase in apoptosis in these cells, as well as in HepG2 cells transfected with the *oeu-*sR-extracted pool (Fig. 5C).

*Oeu*-sR20, *hsa*-miR34a and *oeu*-sR pool transfections induced a significant decrease in the SNAIL protein level in HEPG2 cells and restored the expression of the adhesion protein E-cadherin (Fig. 5D–F), which is crucial in the epithelial morphogenesis process. The scratch test was used to detect the HepG2 cell migration rates at 0 and 72 h after *oeu*-sR20 or *oeu*-small *RNA* extracted pool transfection, confirming the ability of these to counteract the cell migration (Fig. 5G). *Oeu*-sR20 and *oeu*-small-RNA pool transfections were able to prevent hepatic damage associated with lipid accumulation that was induced by TGF-beta treatment^37^, as demonstrated by the significant decrease in lipid accumulation and restoration of the intracellular lipid content as in the control cells (Fig. 5H).

## Discussion

miRNAs are a class of small single-stranded, non-coding RNA present in animals and plants. The ability of miRNAs to regulate post-transcriptional gene expression by binding specific mRNAs relies on two mechanisms: translation repression or target-mRNA degradation, depending on the complementarity degree of the miRNA with the mRNA-binding region.

Although there is a high similarity in the biological function generated from the regulation of transcripts, plant and animal miRNAs exert their control differently. In animals, miRNAs repress gene expression by mediating translation reduction through multiple miRNA-binding sites that are located within the 3′ untranslated region of the target gene. In contrast, most plant miRNAs regulate their targets by directing mRNA cleavage at single sites in the coding regions^1^.

Brennecke and co-workers^38^ reviewed for the first time the minimal requirements for a functional miRNA-mRNA duplex *in vivo* and introduced the concept of “functional sequence homology” related to common post-transcriptional regulations mediated by miRNAs.

Following Zhang’s article^20^, several studies have shown that exogenous plant miRNAs, which are introduced by diet, are taken up into the bloodstream and tissues through the gastrointestinal system. Other studies, which were conducted using next-generation sequencing technology^30^ and *in vivo* mice models^21^ and were supported by computational analysis^22,26,27,32,38,39^, have highlighted a high number of exogenous miRNAs in the human plasma and the potential bioactivity of plant-based dietary miRNAs in other body tissues. Moreover, the robustness and stability of plant miRNAs have also been documented both in high temperatures during cooking and in chemical degradation typically associated with the digestion process^23,24^. Several mechanisms regarding the stability of plant miRNAs have been suggested, such as the presence of a 2′-O-methylation in plant miRNAs, which enhances their stability by protecting them from exonucleolytic digestion and uridylation^40,41^. miRNA carriers such as exosomes, microvesicles, and high-density lipoprotein protect miRNAs from degradation^42–44^. Moreover, some plant extracts and secondary metabolites may protect plant miRNAs from the enzymatic environment of the digestive tract^45,46^.

Additionally, other studies have demonstrated that exogenous plant miRNAs remain functionally active in consumers and that they regulate the post-transcriptional expression of specific human genes^21,30,47^.

The most direct evidence of the therapeutic effects of plant miRNAs has been recently published by Zhang’s group^19^. A plant-derived miRNA, miR2911, is highly stable in the decoction of a Chinese herb honeysuckle; it can be absorbed through the GI tract and delivered via the blood stream to the lungs of the animals, and it protects against influenza virus infections in mice.

Recently, Chin and coworkers published a detailed set of experiments that showed that in an *in vitro* model, plant miR159 suppressed the proliferation of breast cancer (BC) cells^48^. In this manner, by binding and inhibiting its specific human transcripts, *TCF7*, involved in the Wnt signaling pathway, is targeted. This, in turn, induced a substantial reduction of tumor growth and increased apoptosis level in a xenograft BC tumor model.

The identification of plant-derived miRNAs and/or sRs that regulate the human genome expression could be an interesting approach to shed light on the nutritional and functional value of plant foods. Encouraged by these discoveries, we used MirCompare to identify *O*. *europaea*-sRs; these possess functional homology with human miRNAs and the potential to interact with human target genes and modulate their expression.

We selected *oeu*-sR20, *oeu-*sR27 and *oeu-*sR34, which show a sequence homology of 45%, 63% and 45%, respectively, with *h*sa-miR34a. However, to confirm their identity and annotation, when these sRs, which had been previously reported as miRNAs by Yanik *et al*.^31^, were searched within the high performed whole-genome of *O*. *europaea* sp *sylvestris*, which was recently sequenced^40^ and was present in Phytozhome^41^ and NCBI Genome (https://www.ncbi.nlm.nih.gov/genome), the analyses executed with the miRDeep2 tool^42^ resulted in a zero alignment hit. Further studies were then conducted to identify the origin of small RNAs in the genome of var. Sylvestris and var. Farga of *O*. *europaea*. Surprisingly, it emerged that the three sequences that were indicated by Yanik *et al*.^31^ as novel miRNAs are prevalently located in non-coding regions (Table S2), except for one location of sR27 and sR34 that are both annotated as transcript coding sequences (Table S2).

Because it is not clearly known if non-coding regions that contain the sR sequences are expressed or not, we checked and confirmed their presence in the pulp of mature olive drupes via RT-qPCR. Therefore, we advance the hypothesis that small RNAs are fragments of the degradome of non-coding transcripts and mRNAs that are expressed in the tissues during the over-ripening of olive drupes. Their presence in the ripening fruits was also reported by Yanik *et al*.^31^ and was confirmed in the extracts of mature drupes through RT-qPCR analyses in other experiments (data not shown). Recently, evidence has been reported in the literature that in plants and animals, a new class of small RNAs are derived from tRNA^49^, and they exhibit a microRNA-like function^50,51^.

Through these results and evidence from the literature, we hypothesize that a new lateral evolution of small RNA classes might be generated from non-coding regions of the genome that under stress and/or organ development are expressed as reported for *Brassica rapa*^52^. These small RNAs can open a new challenge in the role that they could play in cross-kingdom interaction, and this might be an exciting new research frontier.

Several studies have indicated that *hsa*-miR34a plays a pivotal role in different apoptotic mechanisms by repressing the post-transcriptional expression of *BCL2* and *SIRT1*, which, in turn, induces apoptosis via regulating the mitochondrial pathway and p53 activity, respectively^7–9^.

In various tumors, *hsa*-miR34a is frequently silenced, which suggests an important role of the controller in tumor suppression. The *hsa*-miR34a mimic, which had been already used in different clinical trials, restores the physiological post-transcriptional expression of both its human target genes, *SIRT1* and *BCL2*, promoting an anti-tumoral effect^8,53^. Moreover, the restoration of *hsa*-miR34a reduces cell viability, promotes apoptosis and enhances sorafenib-induced apoptosis and toxicity in HCC via inhibiting *BCL2* expression^54^.

The epithelial to mesenchymal transition (EMT) is considered a key process that is implicated in neoplastic transformation, particularly in invasion and metastatic processes^55^. The strong evidence for EMT in HCC patients demands novel strategies in pathological assessments and therapeutic concepts to effectively combat HCC progression.

SNAIL is directly involved in EMT during tumor progression: SNAIL over-expression is sufficient to induce EMT in many epithelial cell lines and is associated with E-cadherin down-regulation in several mouse and human invasive tumor cell lines^56,57^.

The strategies to increase *hsa*-miR34a could potentially constitute a critical targeted therapy for different tumors in the future. To verify the ability of synthetic sequences of *oeu*-sRs to regulate the post-transcriptional expression of *hsa-*miR34a-specific target, we used lymphoid-monocytoid and hepatocellular tumor cell lines with low expression of *hsa-*miR34a and PBMCs from healthy donors with a normal expression level of *hsa*-miR34a. The obtained results show that synthetic sRs *oeu*-sR20, *oeu*-sR27 and *oeu*-sR34 cause a statistically significant reduction in the SIRT1 and BCL2 protein expression levels with respect to the controls. However, a modulation of the same proteins was not observed in PBMCs despite the efficiency of transfection. As a consequence of BCL2 down-regulation and a rise in BAX level, we detected a remarkable increase in apoptosis levels in tumor cells, whereas PBMCs proved to be unaffected. In hepatic cells, the SNAIL protein reduction after *oeu*-sR20 transfection was associated with a significant increase in E-cadherin expression and a reduction of lipid accumulation. This suggests that plant-derived sRs, through their ability to restore the correct epithelial phenotype, have the potential to counteract liver damage that determines EMT. All obtained data suggest that the transfection of plant sRs that are homologous to *hsa*-miR34a restores the *hsa*-miR34 functions in tumor cells lacking this *hsa-*miRNA. Conversely, cells with the normal expression of endogenous *hsa*-miR34a were observed to be unaffected by exogenous plant sR regulation.

Despite the fact that sequences of plant-derived synthetic sRs show neither complete identity nor high homology to the human miR34a sequence, the results have shown that they exhibit an important “functional homology”, as demonstrated by their capability to modulate the translation of *hsa*-miR34a genomic targets.

Based on the results obtained in this study, we propose to extend this concept to small RNAs (sRs) that, considering their structural sequence, can perform the same functional activity in a cross-kingdom interaction. This consideration was also confirmed via the results obtained from the experiments conducted with sR-mimics and with the pool extracted from drupes, which contained either sRs or miRNAs; among these components of the extracted pool, a high quantity of *oeu*-sR20 and *oeu*-sR34 were present. Accordingly, this research indicated that there are plant small RNAs that are capable of exhibiting antitumor activity upon entering the human cells and performing a fine-tuning function homologous to *hsa*-miRNAs.

Transfection of the olive-extracted pool, as well as the mimics used, induces a decrease in BCL2 and SIRT1 protein expression, enhancement of apoptosis and reduction in the transition from the EMT to MET phenotype.

The application of miRNAs and/or sRs in cancer therapy may involve the administration of these small RNAs with tumor-suppressor function to restore their activities^12,13^.

miRNAs derived from food have been demonstrated to be stable under stress conditions of high temperature during cooking, during enzymatic digestion occurring in the gastrointestinal tract, and in animal serum^23,24^. They are also able to regulate gene expression in organisms that have ingested them^19,20,30,58^, which indicates the possibility that small RNAs act as new bioactive components in herbal remedies.

Oral administration is the only way of application for herbal preparations; however, despite increasing evidence, the mechanism underlying the functional transfer of plant miRNAs across the intestinal barrier remains unclear. Considering the instability of naked RNA, miRNAs in plant materials are, possibly, packaged into protein complexes and/or lipid vesicles and are recognized by a mammalian transport system^23^.

For these reasons, it could be possible to develop a natural, non-toxic nutraceutical compound that contains active tumor suppressor miRNAs and sRs; this would help address the problems of toxicity resulting from chemotherapy^54,59–61^.

The use of edible plants to produce therapeutic small RNAs has ground-breaking potential for clinical applications, and it could be an economical alternative to the current production of synthetic miRNAs. Recent advances regarding the role of miRNAs and sRs, considering the results obtained in this study, could form a basis for further *in vivo* studies to finally understand oncosuppressive functions of plant small RNAs in humans.

Finally, the obtained results support the exposome concept that correlates the environment, as nutrition and epigenetics, with human health: in this work, epigenetic activity is represented by plant miRNAs and their effects on the host after oral intake of plant-based food^62,63^.

So far, it was assumed that plant miRNAs could regulate human cell mRNA translation behavior; this research also indicates that there are plant small RNAs that are capable of exhibiting antitumor activity when entering human cells and performing a fine-tuning function that is homologous to *hsa*-miRNAs.

## Materials and Methods

### Bioinformatics analysis

#### Cross-kingdom analysis

MirCompare^32^ was used to identify small RNA sequences with a functional homology to human miRNAs. The software compared the miR sequences o*f O*. *europaea cv*. Ayvalik^31^ with all *H*. *sapiens* mature miRNAs, which were extracted from miRBase^33^. The parameters used for the analysis are r-value = 0.55 and seed-region threshold = 5 (in accordance with the guidelines provided by the authors).

#### Prediction of small RNA targets

Concerning the prediction of all potential cross-kingdom targets, we assumed that plant sRs regulate host mRNA translation in a manner that is analogous to mammalian functional miRNAs. We used the COMIR^29^ web interface to extract a list of human genes that are putatively regulated by the submitted miRNAs together with a p-value based on scrambled sequences targeting predictions. According to that reported in the COMIR documentation, a total of 20,121 *H*. *sapiens* genes (3′UTR sequences downloaded from the ENSEMBL website) were considered in the analysis. After selecting the significant predictions, Diana TarBase^33^ was used to select the output of COMIR gene-miRNA interactions.

#### sR-mRNA free energy calculation

For the estimation of miRNA-mRNA free energy variations, a custom script was developed that uses the miRanda algorithm^64^ to calculate the free energy of duplex formation. For each plant miRNA sequence, we provided the target mRNAs sequences, in the FASTA format.

#### Validation of initial dataset

To evaluate the nature of the *O*. *europaea* sequences listed by Yanik and co-workers^31^, we extracted the raw sequencing data from SRA52, and we performed an miRDeep242 analysis using the *Olea europaea* var. sylvestris genome^65^ as the reference.

#### Identification of sR location in the genome

To search for the mapping and annotation of all three small RNA, an interrogation of their sequences was run through BLASTn against the whole genome of *O*. *europaea* var. sylvestris deposited in the Phytozome v12.1 database (https://phytozome.jgi.doe.gov/pz/portal.html) and NCBI non-redundant (nr) database. Moreover, we have also interrogated the genome drafts of *O*. *europaea* cv. Farga (http://denovo.cnag.cat/genomes/olive/) and NCBI. From the interrogation, nine hits were produced for the complete sequence of sR20, and eight and 19 hits were produced for the sR-27 and sR-34 sequences, respectively (Table S2). One hit for sR27 and for sR34 was a coding transcript: the first is an annotated transcript identified in the cultivar Picual, and the second is a putative glycine-rich cell wall structural-protein-like transcript located in the chromosome 9 of var. sylvestris.

### Experimental validation *in vitro* and *ex-vivo*

#### Cell culture

Human Jurkat E6-1 lymphoid and human THP1 monocytoid cell lines (American Type Culture Collection, USA) were grown in a suspension culture at a density of 7 × 10^5^ cells/mL and 4 × 10^5^ cells/mL, respectively. The human HepG2 hepatoma-derived cell line was grown in Dulbecco’s modified Eagle’s medium (DMEM) that was supplemented with 10% fetal bovine serum, 100 U/mL penicillin, 100 mg/mL streptomycin and 2 mM L-glutamine (Lonza, USA). Human PBMCs, obtained from healthy blood donors attending at the local blood transfusion unit of Policlinico Tor Vergata in Rome, were separated via density gradient, according to the standard technique by Fycoll Hypaque (Lonza, USA), and were cultured at a density of 10^6^ cells/mL. The cell lines and PBMCs were cultured in Roswell Park Memorial Institute (RPMI) 1640 (Invitrogen, USA) that was supplemented with 10% fetal bovine serum (FBS, Invitrogen USA), 2 mM glutamine (Hyclone, UK), 50 U/mL penicillin and 50 U/mL streptomycin (Hyclone, UK). All the cell lines were cultured at 37 °C under a humidified 5% CO_2_ atmosphere.

#### Transfection

THP1 monocytoids, Human Jurkat E6-1 lymphoid cells and PBMCs were all transfected via the lipofectamine method (Hi-Fect, Qiagen German, HF) using 5 nM synthetic mimic human miR34 (*hsa-*miR34) or a scramble of *hsa*-miR34a and three homologous vegetal mimics (*oeu*-sR20, *oeu-*sR27 and *oeu-*sR34) (Table S3, INVITROGEN, USA) in accordance with the manufacturer’s instruction (miRNA mimic and inhibitor experiment protocols®, Qiagen, Italy). *Oeu*-sR mimics that were tagged with fluorescein isothiocyanate (FITC) were also used as the transfection control.

The cells were harvested 72 hours after transfection and were characterized for the efficiency of transfection through fluorescence microscopy and flow cytometry analysis and for the effect of *hsa*-mir34 and plant sRs on specific target genes via western blot analysis.

#### Fluorescence microscope analysis

*Oeu*-sRs green positive cells, that were transfected for 72 hours with FITC-tagged *oeu*-*s*Rs mimics, were observed under a fluorescence microscope (Evos Floid Cells Imaging Station, ThermoFisher Scientific, USA). In some experiments, to evaluate the localization of *oeu*-sRs in the cells, the cells were harvested, fixed, permeabilized with 70% ethanol and incubated with anti-human calreticulin for 1 hour and Alexa-Fluor-647-conjugated secondary antibody (ThermoFisher Scientific).

#### Cell viability assays

The percentage or absolute number of dead/living cells was evaluated using the trypan blue dye exclusion test (Euroclone S.p.A. ITA).

#### Apoptosis assays

Seventy-two hours after transfection, apoptosis was assessed through flow cytometry analysis (Cytoflex, Beckman Coulter, USA) of isolated nuclei that were stained with propidium iodide, as previously described^66^.

#### Intracellular BCL2 staining

BCL2 intracellular expression was evaluated via flow cytometry analysis. After 72 hours, the transfected cells were harvested, fixed, permeabilized with 70% ethanol and incubated with PE-conjugated anti-human BCL-2 (BD Biosciences, USA). The stained cells were analyzed via Cytoflex (Beckman Coulter, USA) and the Cytexpert 1.2 software (Beckman Coulter, USA).

#### Analysis of lipid accumulation

Seventy-two hours after transfection, for the analysis of lipid accumulation, the cells were suspended with BODIPY® fluorescent probe (Thermo Fisher Scientific, USA) and were incubated at 4 °C for 30 minutes. Flow cytometry analysis was performed via Cytoflex (Beckman Coulter, USA) and Cytexpert 1.2 software (Beckman Coulter, USA).

#### *In vitro* scratch assay

HepG2 cells in the presence or absence of the *oeu*-extracted pool or *oeu*-sR20 were plated, and after 24 hrs, a 10 μL pipette tip was used to make a scratch in the middle of the plate. The cells were allowed to migrate, and images of the scratch width were taken at 72 hours after the initial scratch.

### mRNA isolation, cDNA synthesis and qRT-PCR for the expression of SIRT1 and BCL2 genes

Real-time PCR was performed on a CFX96 Real-Time System instrument (Bio-Rad, CA, USA) with gene-specific primers (BIOFAB, Rome, Italy) (Table S4) and SYBR Green protocol.

Total RNA was isolated using the TRIzol reagent (Invitrogen, CA). The integrity of total RNA was determined via 1% agarose gel electrophoresis. cDNA synthesis was performed using high-capacity cDNA Reverse Transcription Kits (Applied Biosystem by Life Technologies NY, USA Invitrogen, CA) using 1 μg of total RNA as the template, according to manufacturer’s instructions. Real-time PCR reaction was performed as previously described^67^. Quantification was performed using the threshold cycle (Ct) comparative method according to the MIQE guidelines^68^.

#### Plant-small-RNA pool extraction and transfection

Total *O. europaea* small pool (containing *oeu*-miRs and *oeu-*sRs) was extracted from 50 mesocarp tissue mature drupes using NucleoSpin miRNA in accordance with the manufacturer’s instruction (NucleoSpin miRNA experiments protocols®, MACHEREY-NAGEL, Germany) and were transfected into the Jurkat cell line, as described above.

#### Experimental validation of conserved plant miRNAs and small RNA via Quantitative Real-Time PCR (qRT-PCR)

Total miRNAs were purified from plant tissues obtained in the previous step. The presence of most of the conserved plant miRNAs (Table S5) in *Olea europaea* drupe extract was evaluated via qRT-PCR, as widely reported in Gismondi *et al*.^69^. In brief, cDNA was synthesized using a specific reverse transcription kit for microRNAs (miRCURY LNA Universal RT microRNA PCR, Synthesis Kit II; EXIQON), according to the manufacturer’s guidelines. To verify the absence of nucleases in the reaction and to evaluate the efficiency of retro-transcription and RT-qPCR amplification, 10^8^ copies of a synthetic spike-in control miRNA (UniSp6, EXIQON) were added to each RNA sample before conversion to cDNA. RT-qPCR was performed in a 10 µL reaction volume, which included 20 ng of cDNA, 50% SYBR green (ExiLENT SYBR® Green master mix, EXIQON) and 1 µL of a mixture that contained pre-designed PCR primers specific for microRNA amplification (microRNA LNA PCR primer sets, EXIQON). RT-qPCR assay was performed using a Bio-Rad (IQ5) thermocycler. Amplification parameters were set as recommended by the instruction manual of EXIQON pre-designed primers.

The same experimental protocol was used to analyze the expression of *hsa*-miR34a in different tumor cell lines compared to PBMCs. Relative expression levels of *hsa*-miR34a were quantified by using the 2^−ΔΔCt^ method, and 18 S rRNA was used as a housekeeping gene.

#### Western blot analysis

Aliquots of 3 × 10^6^ cells, subjected to different experimental conditions, were lysed and processed for western blot analysis, as previously described^54^. The primary antibodies used were rabbit monoclonal antibodies directed against SIRT1, BCL2 and SNAIL proteins; mouse monoclonal anti-BAX; E-cadherin and calreticulin (used for microscope images, see above); and goat monoclonal anti-human beta-actin (all from Santa Cruz Biotechnology, CA USA). The secondary antibodies used were anti-goat, anti-mouse and anti-rabbit IgG chain specific conjugated to peroxidase (Calbiochem, Merck Millipore, Darmstadt, Germany) and anti-rabbit IgG-PE for microscopy images (Calbiochem).

#### Statistical Analysis

Data analysis was performed using the SPSS statistical software system (Chicago, IL, USA). Comparison of means of FL1-positive cells, protein levels, cell vitality, percentage of apoptosis and SIRT1 and BCL2 positive cells in response to transfection were all conducted using the t-test; p-value < 0.05 (*), p < 0.01 (**) or p < 0.001 (***) were considered significant. For non-parametric correlation, the Pearson correlation coefficient was calculated.

## Electronic supplementary material

Supplementary Data
