# Supplementary material for: Olea europaea small RNA with functional homology to human miR34a in cross-kingdom interaction of anti-tumoral response
Source: Sci Rep. 2018 Aug 17;8:12413. doi: 10.1038/s41598-018-30718-w (PMC6098056; doi:10.1038/s41598-018-30718-w)
Supplement: Supplementary file 1 — Supplementary Data [file 41598_2018_30718_MOESM1_ESM.pdf]

Olea europaea small RNA with functional homology to human miR34a in cross-kingdom interaction of anti-tumoral response

Antonella Minutolo[1+], Marina Potestà[1+], Angelo Gismondi[1+], Stefano Pirrò[1,2], Marco Cirilli[3], Fabiano Gattabria[3], Andrea Galgani[2,4], Libera Sessa[1], Maurizio Mattei[4], Antonella Canini[1], Rosario Muleo[3], Vittorio Colizzi[1,2], Carla Montesano[1*]

**S3 Table: Synthetic miRNA sequences following Yanik's nomenclature (Yanik 2013)**	
**Synthetic miRNA name**	**Synthetic miRNA sequence**
hsa-miR-34	3’-UGUUGGUCGAUGUCUGUACGGU
hsa-miR-34 scramble	3’-UGUGUCGGUGGUUCGAGUCUAU
oeu-miR-20	3’-ACAGUGGUGGUGGUGGUGGUGGU
oeu-miR-27	3’-UGGUGGCGGUGGCGGUGGCGGU
oeu-miR-34	3’-GGUGGCGGUGGAGGUGGAGGU

**S4 Table: SIRT1, BCL-2 and Beta actin primers**		
Sirt-1	5'-TTCGCTCTTTTCCTCCGTCC-3'	3'-CAGCGTGTCTATGTTCTGGGT-5'
Bcl-2	5’-TCCCTCGCTGCACAAATACTC-3’	3’-ACGACCCGATGGCCATAGA-5’
Beta actin	5’-GCACTCTTCCAGCCTTCC-3’	3’-AGGTCTTTGCGGATGTCCAC-5’

**S5 Table: miRNA sequences primers following Yanik's nomenclature (Yanik 2013)**	
**Plant miRs**	**5’ → 3’ sequences**
miR 20	UGGUGGUGGUGGUGGUGGUGACA
miR34	UGGAGGUGGAGGUGGCGGUGG
miR156a	CUGACAGAAGAGAGUGAGCAC
miR 159a	UUUGGAUUGAAGGGAGCUCUA
miR159c	UUUGGAUUGAAGGGAGCUCCU
miR162a	UCGAUAAACCUCUGCAUCCA
miR166i	UCGGACCAGGCUUCAUUCCCCC
miR167-5p	UGAAGCUGCCAGCAUGAUCUU
miR171a	UGAUUGAGCCGUGCCAAUAU
miR395a	CUGAAGUGUUUGGGGGAACUC
miR396c	UUCCACAGCUUUCUUGAACGU
miR482b	UCUUUCCUAUCCCUCCCAUUCC
miR 858b	UUCGUUGUCUGUUCGACCUUG
miR2118a	CUACCGAUGCCACUAAGUCCCA

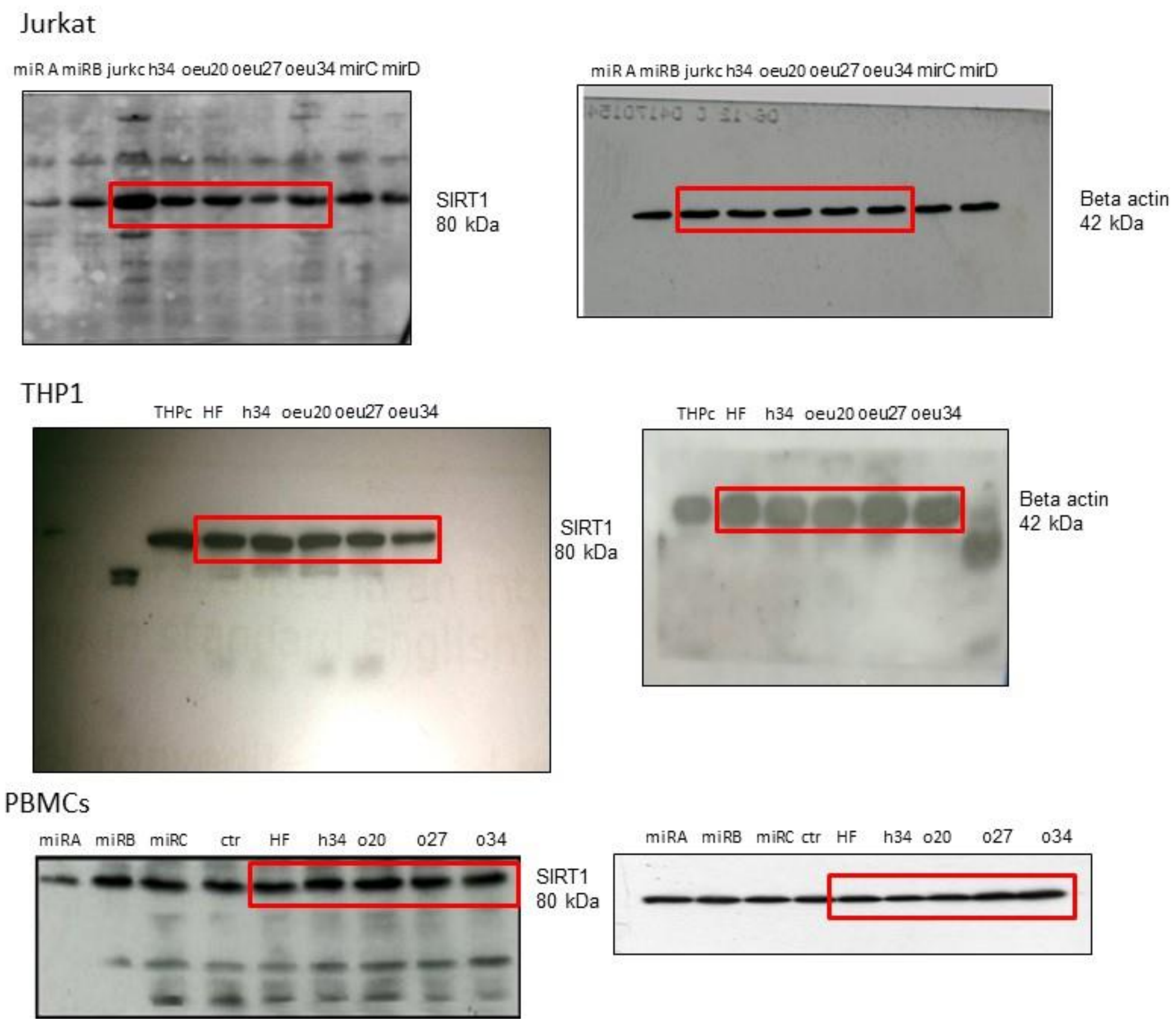

Figure S1. Picture of Biomax FILM (Kodak) of HepG2 cells transfected for 72hrs with High Fect (vehicle) and 5nM of *hsa*-miR34, *oeu* sR20, *oeu*-sR pool. Five minutes of exposition for Jurkat and THP1 cells, 2 minutes for PBMCs. In the red panel the cropped image represented in the Figure 2B of the Article. SIRT1 and the relative beta Actin protein expression are represented. For SIRT1 and its beta Actin a 10% gel was used. MiR-A, -B, -C and D are outside the results of this work.

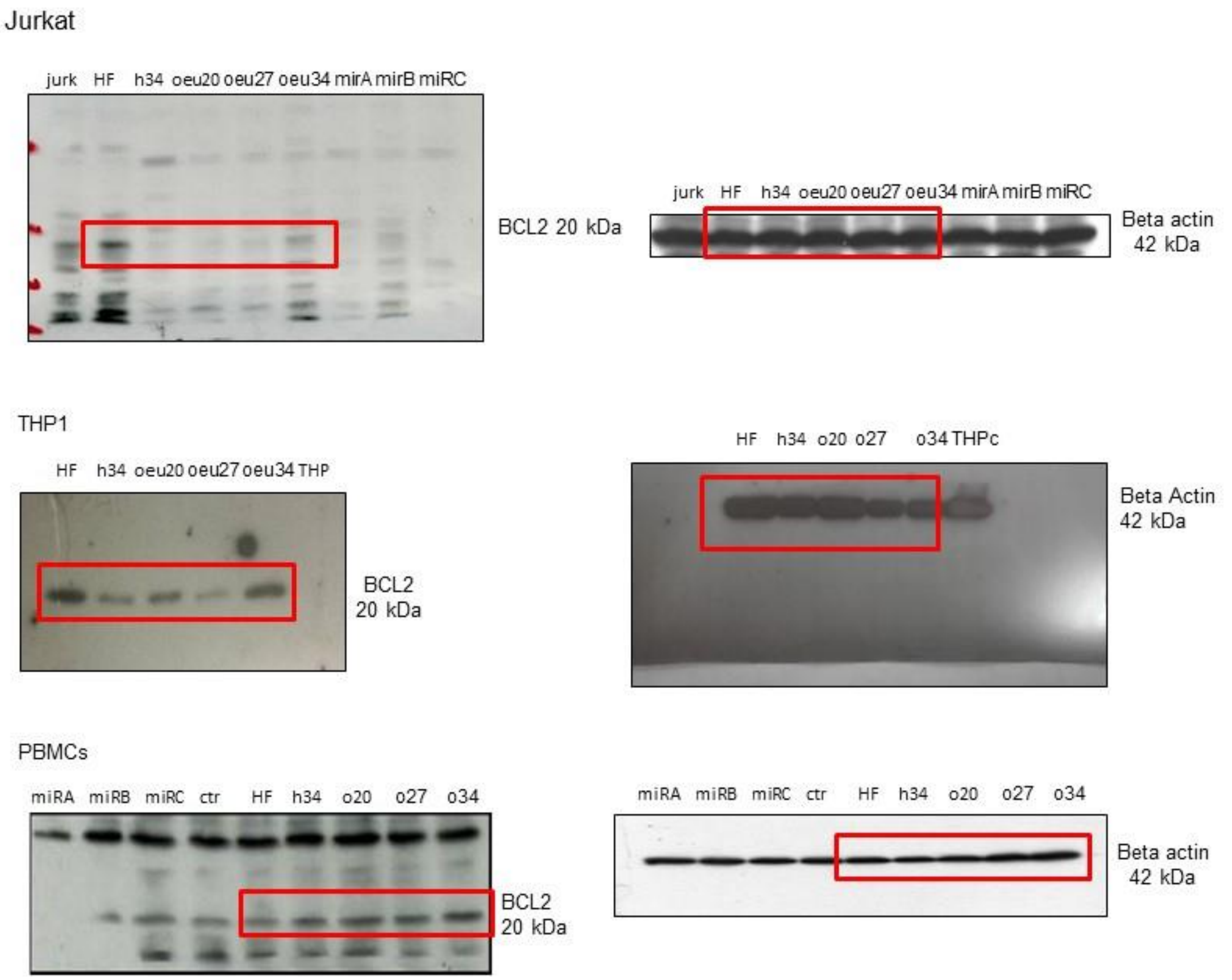

Figure S2. Picture of Biomax FILM (Kodak) of HepG2 cells transfected for 72hrs with High Fect (vehicle) and 5nM of *hsa*-miR34, *oeu* sR20, *oeu*-sR pool. 5 minutes of exposition for Jurkat and THP1 cells, 2 minutes for PBMCs . In the red panel the cropped image represented in the Figure 2G of the Article. BCL-2 and the relative beta Actin protein expression are represented. For BCL-2 and its beta Actin a 10% gel was used. MiR-A, -B, and C are outside the results of this work.

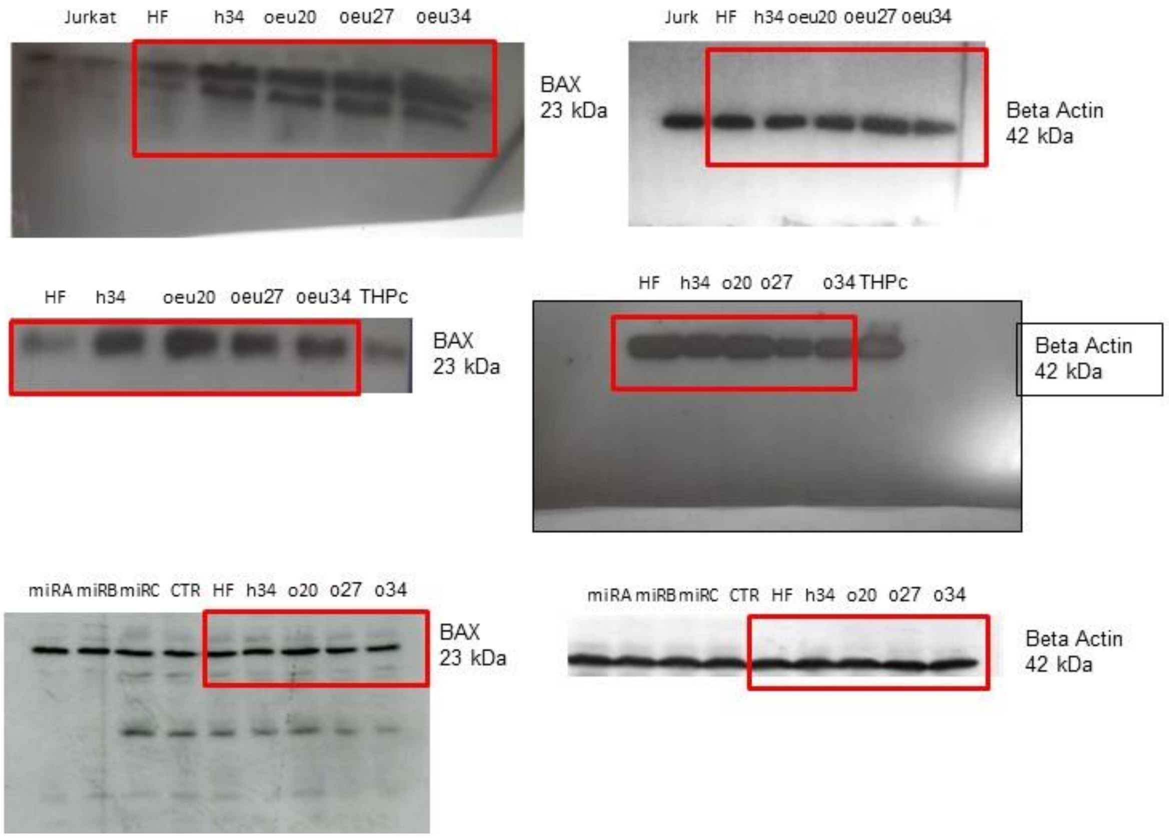

Figure S3. Picture of Biomax FILM (Kodak) of HepG2 cells transfected for 72hrs with High Fect (vehicle) and 5nM of *hsa*-miR34, *oeu* sR20, *oeu*-sR pool. 5 minutes of exposition for Jurkat and THP1 cells, 2 minutes for PBMCs. In the red panel the cropped image represented in the Figure 3D of the Article. BAX and the relative beta Actin protein expression are represented. For BAX and its beta Actin a 15% gel was used.

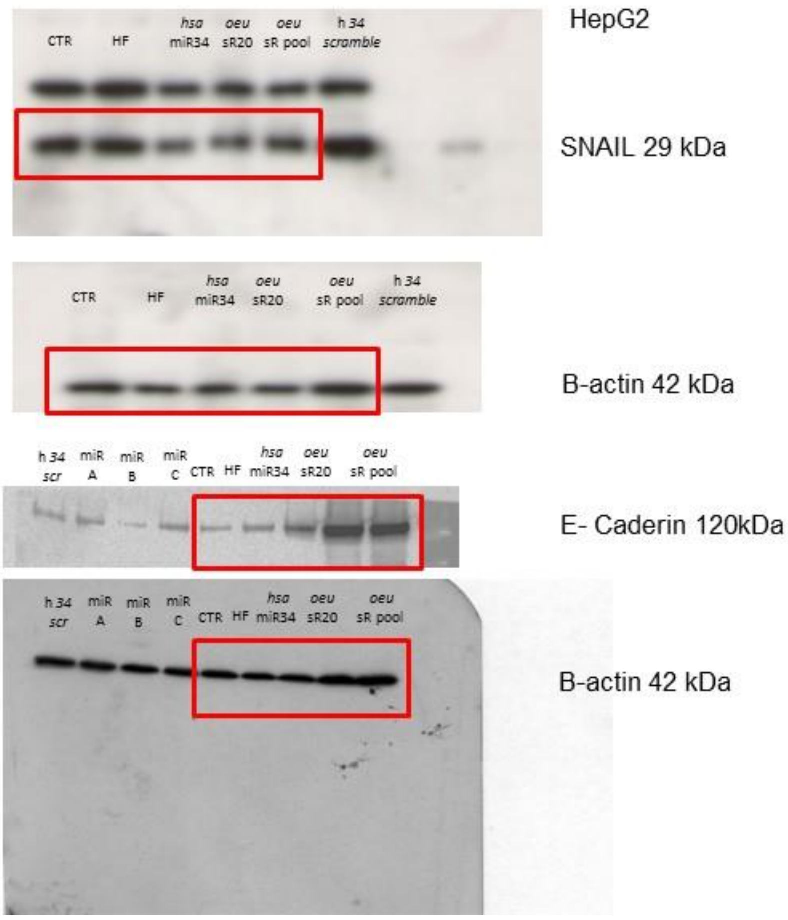

Figure S4. Biomax FILM (Kodak) of HepG2 cells transfected for 72hrs with High Fect (vehicle) and 5nM of *hsa*-miR34, *oeu* sR20, *oeu*-sR pool and a scramble of *hsa*-miR34 as negative control. 2 minutes of exposition. In the red panel the cropped image present in the 5D figure of the Article. SNAIL, E cadherin and the relative beta Actin protein expression are represented. For SNAIL and its beta Actin a gel (15%) was prepared and 10% for E Cadherin and the relative beta actin
